# Tyr–Mpa Proximity-Induced Atypical *tert*-Butyl Intramolecular Migration in Desmopressin: Mechanistic Elucidation of a Site-Selective Impurity and Its Suppression Strategy

**DOI:** 10.1021/acsomega.6c07280

**Published:** 2026-08-27

**Authors:** Xin Lu, Fangfang Yuan, Xiaojie Han, Tingting Qin, Qianqian Wang, Zengli Li, Chuanjun Ge, Wenqing Shi, Haijiao Bai, Pei Wang

**Affiliations:** † 682997 Tianjin Institute for Drug Control, No. 98, Guizhou Road, Tianjin 300070, China; ‡ State Key Laboratory of Medicinal Chemical Biology, 12538 Nankai University, Tianjin 300350, China; § Anhui Anke Biotechnology (Group) Co., Ltd, No. 9 Haiguan Road, Hefei, Anhui Province 230088, China

## Abstract

Desmopressin, a synthetic analog of vasopressin widely used in the treatment of diabetes insipidus and bleeding disorders, is a representative disulfide-containing peptide therapeutic prepared by Fmoc-based solid-phase peptide synthesis (SPPS). Its unique sequence architecture, particularly the spatial proximity between the N-terminal mercaptopropionic acid residue Mpa(1) and the Tyr(2) residue, provides a distinct structural environment for investigating sequence-dependent side reactions during peptide manufacturing. The *tert-*butyl (*t-*Bu) protecting group is widely employed for side-chain protection in Fmoc-based solid-phase peptide synthesis (SPPS). However, under certain reaction conditions, the highly reactive *t-*Bu^+^ carbocation generated during acidic deprotection can participate in nonspecific alkylation, leading to challenging process-related impurities. In this study, we investigated a unique and highly site-selective +56 Da impurity observed during side-chain deprotection. High-resolution MS/MS analysis unambiguously identified this species as a site-selective product arising from the migration of *t-*Bu from the Tyr(2) side chain to the thiol group of Mpa(1). Based on the structural features of desmopressin, in particular, the spatial proximity between Tyr(2) and Mpa(1), we propose that the formation of this impurity most plausibly proceeds via a vicinal-thiol-induced intramolecular migration mechanism, which competes significantly with the conventional diffusion-controlled carbocation intermediate intermolecular pathway. Strengthening the scavenger conditions failed to eliminate this impurity, further supporting the intramolecular migration mechanism induced by vicinal thiol. This finding accounts for the observed site selectivity that cannot be explained by the conventional carbocation intermediate pathway alone. Guided by these mechanistic insights, we re-evaluated the necessity of Tyr side-chain protection and proposed a “source-elimination” strategy. Experimental validation demonstrated that the use of unprotected Fmoc-Tyr-OH (free phenolic hydroxyl) completely suppressed the formation of the *t-*Bu migration impurity and improved the crude peptide purity to 93.5%. Overall, this study elucidates the formation mechanism of *t-*Bu migration impurities and provides a broadly applicable, mechanism-driven strategy for process optimization in solid-phase peptide synthesis. While this study focuses on the desmopressin system, the findings highlight a proximity-driven reactivity pattern that may be relevant to other thiol-containing peptide sequences.

## Graphical abstract

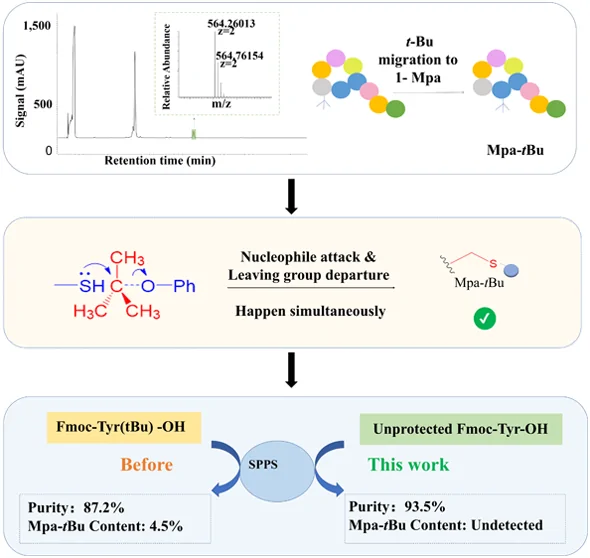

## 1 Introduction

Solid-phase peptide synthesis (SPPS) has become the cornerstone technology for the preparation of modern peptide therapeutics owing to its operational simplicity, high degree of automation, and precise control over sequence assembly.^1^ Nevertheless, SPPS still faces numerous practical challenges. Side reactions may occur during deprotection and resin cleavage, leading to the formation of structurally diverse and process-dependent impurities.^2,3^

Traditionally, such impurities can be removed via preparative chromatography. However, this downstream strategy exhibits significant limitations during the scale-up. The chromatographic resolution between impurities and the target peptide may vary unpredictably with process scale, loading capacity, or batch-to-batch fluctuations. Additional purification steps also impose substantial increases in solvent consumption and processing time.^4^ More critically, product loss during purification is inevitable, thereby compromising the overall yield and economic efficiency. Consequently, minimizing or eliminating impurities through upstream process optimization represents a more sustainable and scalable approach, aligning with the ongoing trend in peptide manufacturing toward higher efficiency, lower cost, and greener production paradigms.^5,6^

Among the many steps in SPPS, the removal of side-chain protecting groups represents one of the most critical challenges in process control. This step is typically conducted under strongly acidic conditions, where highly reactive intermediates—such as carbocations or radical species—are transiently generated. Simultaneously, nucleophilic or electrophilic functional groups on the partially deprotected peptide are exposed to these high-energy environments. Together, these factors create a favorable setting for nonspecific alkylation driven by carbocationic or radical pathways. In practical process development, common mitigation strategies include tuning the ratios of cleavage reagents, scavengers, and reductants to balance deprotection efficiency against suppression of side reactions, and incorporating functional auxiliary additives when needed.^7^

Desmopressin is a synthetic analogue of vasopressin widely used in the treatment of diabetes insipidus and bleeding disorders. Structurally, it contains a modified N-terminal mercaptopropionic acid (Mpa) residue and a tyrosine residue at position 2, which together creates a unique spatial arrangement involving a thiol and a phenolic functional group.This structural feature provides a relevant model system for investigating proximity-driven side reactions during peptide synthesis.

The *t-*Bu group is one of the most widely used protecting groups for masking the phenolic hydroxyl of tyrosine (Tyr). Removal of the *t-*Bu group under strongly acidic conditions proceeds predominantly through a classical carbocation intermediate pathway.^8^ It is well established that *t-*Bu, as an acid-labile protecting group, typically undergoes protonation of the phenolic oxygen, followed by heterolytic cleavage to generate a *t-*Bu^+^ that is subsequently quenched. However, if scavenger levels are inadequate, *t-*Bu^+^—as a highly potent electrophile—may undergo nonspecific alkylation with nucleophilic side chains exposed on the peptide, including residues susceptible to electrophilic attack such as Cys, Trp, and Arg.^9^

In the desmopressin system investigated in this study, we identified a *t-*Bu-migration impurity that exhibits an unexpected level of site selectivity that deviates from the classical nonselective behavior typically associated with a conventional carbocation intermediate pathway. Only a single, site-specific +56 Da impurity was observed in our system, thereby suggesting the involvement of an alternative, more selective reaction pathway.^10,11^

Further investigation revealed that the vicinal arrangement of Tyr and Mpa in desmopressin provides a feasible intramolecular geometric context for the unexpected reaction. The thiol of Mpa, being a neutral nucleophile, is capable of engaging in a vicinal-thiol-induced intramolecular nucleophilic substitution onto the *tert*-Bu moiety under strongly protic and polar cleavage conditions. This process competes with the conventional diffusion-controlled carbocation intermediate intermolecular processes and induces highly site-selective *t-*Bu migration. These findings not only highlight that analogous nonclassical migration events may warrant attention in peptide systems containing thiol functionalities but also suggest that sequence-specific geometry can override classical protecting group logic.

Building upon this mechanistic insight, we further reassessed the necessity of Tyr side-chain protection to prevent the formation of a reactive substitution window altogether. Ultimately, we demonstrated that employing unprotected Fmoc-Tyr-OH completely suppresses the *tert*-Bu migration impurity and markedly improves crude-peptide purity.

This study aims to provide a systematic solution for controlling *tert*-Bu migration impurities through a closed-loop workflow comprising structural identification, mechanistic elucidation, process optimization, and experimental validation. Moreover, the findings offer a generalizable paradigm for mechanism-driven process development in complex peptide syntheses.

## 2 Materials and Methods

### 2.1 Chemicals and Materials

All reagents used in the deprotection procedures were of analytical grade. Trifluoroacetic acid (TFA), triisopropylsilane (TIS), ethanedithiol (EDT), thioanisole (TAO), phenol (PhOH), 3-mercaptopropionic acid (Mpa), tris­(2-carboxyethyl)­phosphine (TCEP), and iodoacetamide (IAM) were purchased from Aladdin (Xi’an, China). Acetonitrile (ACN), sodium dihydrogen phosphate, and phosphoric acid for HPLC analysis were of analytical grade and supplied by Aladdin (Xi’an, China). Reagents used for UPLC-HRMS analysis were LC–MS grade; ACN, formic acid (FA), TCEP, and IAM were purchased from Sigma-Aldrich (Shanghai, China). Purified water used throughout the study was obtained from Wahaha (Hangzhou, China). 4-(2′,4′-Dimethoxyphenyl-Fmoc-aminomethyl)-phenoxyacetamido-aminomethyl resin (Rink amide AM resin, substitution degree 0.3–1.0 mmol/g) was purchased from Polypeptide Group (Sweden). The following Fmoc-protected amino acids were sourced from GL Biochem Ltd. (Shanghai, China): Fmoc-Gly-OH, Fmoc-D-Arg­(Pbf)-OH, Fmoc-Pro-OH, Fmoc-Cys­(Trt)-OH, Fmoc-Asn­(Trt)-OH, Fmoc-Gln­(Trt)-OH, Fmoc-Phe-OH, Fmoc-Tyr-OH, Mpa­(Trt)-OH, and Fmoc-Tyr­(*t-*Bu)-OH. *N*,*N*’-Diisopropylcarbodiimide (DIC, 99%), 1-hydroxybenzotriazole (HOBt), piperidine, *N*,*N*-dimethylformamide (DMF), and hydrogen peroxide (H_2_O_2_) were all supplied by Sigma-Aldrich.

### 2.2 HPLC Conditions

The separations of crude peptides were carried out on a 150 mm × 4.6 mm (i.d.) column packed with 2.7 μm particle sized octadecyl silica from Agilent company (Santa Clara, CA) at 0.8 mL/min and 35 °C using an Ultimate 3000 high performance liquid chromatography from Thermo Fisher Scientific (Dallas, TX). The mobile phase A was water containing 20 mM phosphate, pH adjusted to 4.0 with phosphoric acid. The mobile phase B was 50% mobile phase A and 50% acetonitrile. The eluent gradient was as follows: 10% B (initial–2.0 min), 10–36% B (2.0–25.0 min), 36–70% B (25.0– 40 min), 70–100% B (40–45 min), 100% B (45–50 min), 100–10% B (50.0–51.0 min), and 5% B (51.0–60.0 min). The chromatograms of the analytes obtained are shown in Figure 1.

**Figure 1:**
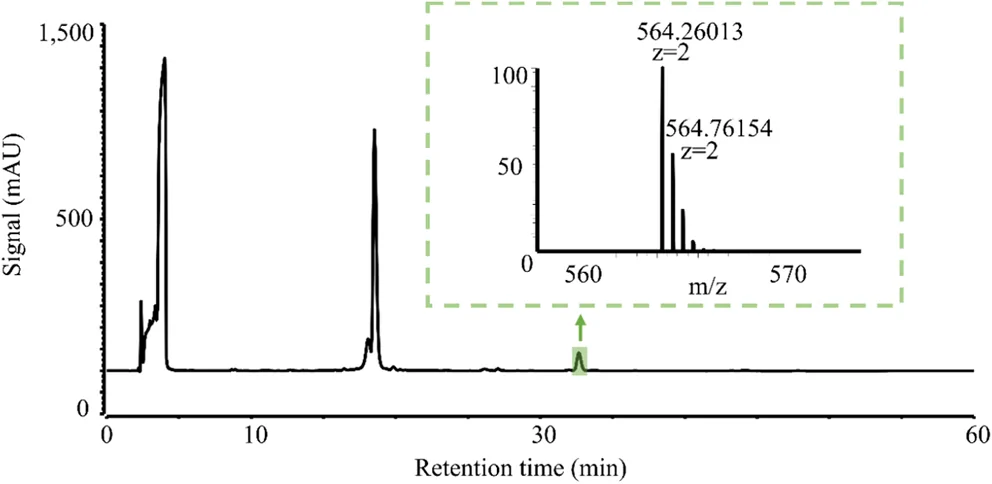
Chromatogram of crude peptide and mass spectrum of target impurity.

### 2.3 UPLC-MS/MS Conditions

The separations were carried out on a 100 mm × 2.1 mm (i.d.) column packed with 1.7 μm particle sized octadecyl silica from Agilent company (Santa Clara, CA) at 0.3 mL/min and 50 °C using a Vanquish Ultra Performance Liquid Chromatography from Thermo Fisher Scientific (Dallas, TX). The mobile phase A was water containing 0.1% formic acid. The mobile phase B was acetonitrile. The eluent gradient was as follows: 5% B (initial–2.0min), 5–90% B (2.0–9.0 min), 90% B (9.0–11.0 min), 90–5% B (11.0–12.0 min), and 5% B (12.0–14.0 min). The chromatograms of the analytes obtained are shown in Figure 1.

The detection was conducted using Orbitrap Exploris 480 Mass Spectrometry (ThermoFisher) with positive heated electrospray ionization mode. The ionization was performed at ion spray voltage of 3200 V, sheath gas of 35 Arb, Aux gas of 10 Arb, sweep gas of 0 Arb, ion transfer tube temperature of 320 °C, vaporizer temperature of 350 °C. The ddMS2 experiments were performed at the resolution of 15,000, HCD Collision Energy of 35 eV, RF lens of 50%, AGC target of standard. For peptide sequencing, data process was performed in Thermo Scientific BioPharma Finder 4.1.

### 2.4 Synthesis of Desmopressin Acetate

#### 2.4.1 Preparation of Desmopressin Peptidyl Resin is shown in Supporting Information.

#### 2.4.2 Global Deprotection and Resin Cleavage

The fully assembled desmopressin peptidyl resin was treated with cleavage cocktail (100 mL) consisting of TFA/TIS/TAO/PhOH/H_2_O = 80:6:5:5:4 (v/v/v/v/v) supplemented with DTT (2 equivalents). The cleavage reaction was carried out at 25 °C for 120 min under gentle agitation. After completion, the cleavage solution was filtered to remove the spent resin and slowly poured into cold diethyl ether (500 mL) with vigorous stirring to precipitate the crude peptide. The resulting suspension was filtered, and the solid was washed five times with diethyl ether. The crude peptide was then dried under vacuum in a desiccator to afford 2.105 g of crude product.

#### 2.4.3 Complete process flow diagram is shown in Figure S1

## 3 Results and Discussion

### 3.1 Observation of a Distinct +56 Da Impurity and Preliminary Hypothesis of Its Origin

Under the standard cleavage conditions (TFA/ triisopropylsilane (TIS) /thioanisole (TAO)/PhOH/H_2_O = 80/6/5/5/4 with added DTT, 25 °C, 2 h), a prominent impurity was consistently observed in the crude HPLC profile of the target peptide. (In addition, the front peak (retention time: 18.830 min) observed preceding the main peak (retention time: 19.203 min) corresponds to an oxidized form of the target peptide. As the subsequent step involves oxidation of the main component, this species becomes the desired product and is therefore not considered an impurity at this stage.) This impurity appeared at approximately 4.5%, substantially exceeding the acceptable level for routine SPPS processes and markedly higher than any other coexisting byproducts. As a consequence, the crude purity decreased to 77.52%, leading to a significant reduction in the overall synthetic yield. High-resolution mass spectrometry revealed that the impurity exhibited a molecular mass 56.06266 Da greater than that of the target peptide (See Figure 1). This mass shift is consistent with the involvement of a *t-*Bu group (theoretical molecular weight 56.06260), consistent with a transformation originating from the Tyr(2) side-chain protecting group.

In Fmoc-SPPS, the *t-*Bu protecting group is removed under strongly acidic conditions through a classical carbocation intermediate pathway, which may generate two general classes of process-related impurities.^12^ (1) Incomplete deprotection—insufficient acidity, inadequate reaction temperature, or shortened reaction time may result in retention of the original *t-*Bu protecting group on Tyr; (2) Nonselective alkylation—when scavengers are insufficient, the *t-*Bu^+^ may act as a potent electrophile, reacting with exposed nucleophilic side chains such as Mpa, Cys, or Arg, leading to a variety of nonspecific alkylated impurities.

### 3.2 Strengthening the Cleavage Conditions Fails to Reduce the Impurity Level: Excluding Incomplete *t*-Bu Deprotection as the Source

If the +56 Da impurity originated from incomplete cleavage of the Tyr side-chain *t-*Bu protecting group, then applying more forcing acidic conditions should reduce its abundance. To test this hypothesis, we intensified the original cleavage cocktail (TFA/TIS/TAO/PhOH/H_2_O = 80:6:5:5:4 with 2 eq DTT, 25 °C and 2 h) and systematically evaluated several modified conditions, including (1) A stronger acidic system, increasing TFA content from 80% to 90% (TFA:TIS: ethanedithiol (EDT): PhOH: H_2_O = 90:4:2:2:2 with added DTT and TCEP); (2) Elevated reaction temperature, increasing from 25 to 30 °C; and (3) Extended reaction time, lengthening from 2 to 2.5 h.

The resulting crude products were analyzed by HPLC. The chromatograms are shown in Figure 2 and the data are summarized in Table 1. The corresponding raw chromatograms are provided in the Supporting Information (Figures S2–S4).

**Figure 2:**
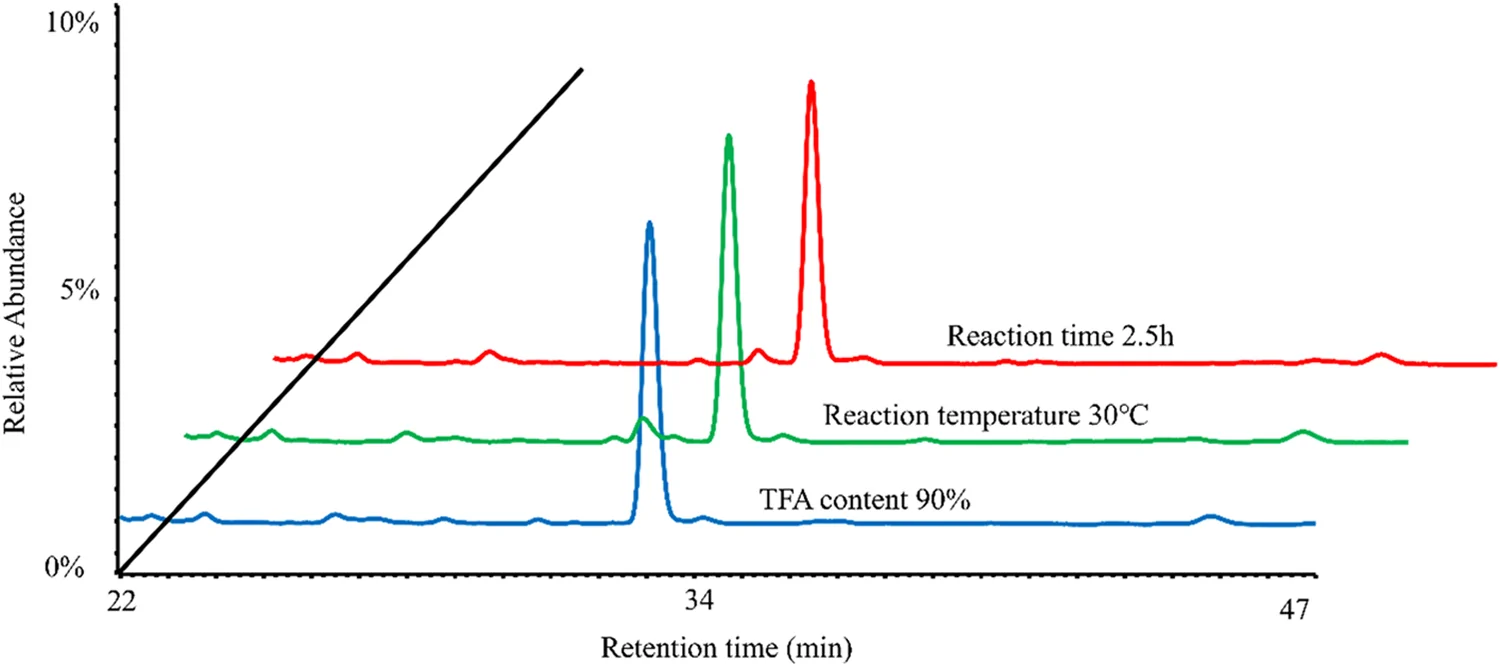
Comparative chromatographic profiles of the crude peptide obtained after process modifications, including increased TFA content, elevated reaction temperature (30 °C), and extended reaction time (2.5 h).

**Table 1:** Impurity Content and Crude Peptide Purity in Strengthening Cleavage Conditions

factors	Mpa(*t*-Bu) impurity content (%)	crude peptide purity (%)
TFA content 90%	6.97	86.0
reaction temperature 30 °C	6.36	84.7
reaction time 2.5 h	5.84	82.6

Despite substantially strengthened cleavage conditions, the impurity level did not decrease. Instead, a slight increase was observed in all cases, and the overall crude purity showed no meaningful improvement. These findings are inconsistent with the typical behavior of incomplete *t*-Bu deprotection and are consistent with the fact that the impurity does not arise from insufficient cleavage of the Tyr protecting group. Rather, its formation must involve a mechanistic pathway distinct from that of conventional *t-*Bu removal.

### 3.3 Structural Elucidation of the Impurity by MS/MS Fragmentation: *t*-Bu Migration to Mpa(1)

The standard peptide cleavage cocktail is dominated by trifluoroacetic acid (TFA), a highly acidic and strongly protic polar medium. Under such conditions, *t-*Bu–based protecting groups (e.g., the phenolic *t-*Bu ether on Fmoc-Tyr­(*t-*Bu)-OH) are typically removed through a classical carbocation intermediate pathway. In this pathway, the *t-*Bu–O–Ar moiety is first protonated at the phenolic oxygen, significantly weakening the C–O bond. Subsequent heterolysis of this bond generates a *t-*Bu^+^ and liberates the free phenol. The resulting *t-*Bu^+^ is rapidly trapped by nucleophiles present in the scavenger system (e.g., water, thiols, or other nucleophilic additives), thereby terminating the reaction.

This process represents a canonical carbocation intermediate-type deprotection mechanism with the following defining features: (1) bond cleavage precedes nucleophilic capture; (2) formation of a carbocation intermediate leads to poor positional or regiochemical selectivity, consistent with the formation of comparable levels of Mpa­(*t-*Bu), Cys­(*t-*Bu), and Arg­(*t-*Bu) adduct impurities; and (3) the product distribution is highly sensitive to the concentration of scavengers.

To elucidate the precise structure of the +56 Da impurity, which appeared as the sole significant byproduct in the chromatogram, we performed high-energy collision dissociation (HCD) MS/MS analysis on the corresponding peak.^13^ The fragment ion pattern revealed that all *t-*Bu–containing diagnostic ions unambiguously corresponded to a modified form of Mpa(1). MS/MS spectrum of the target impurity is shown in Figure 3.

**Figure 3:**
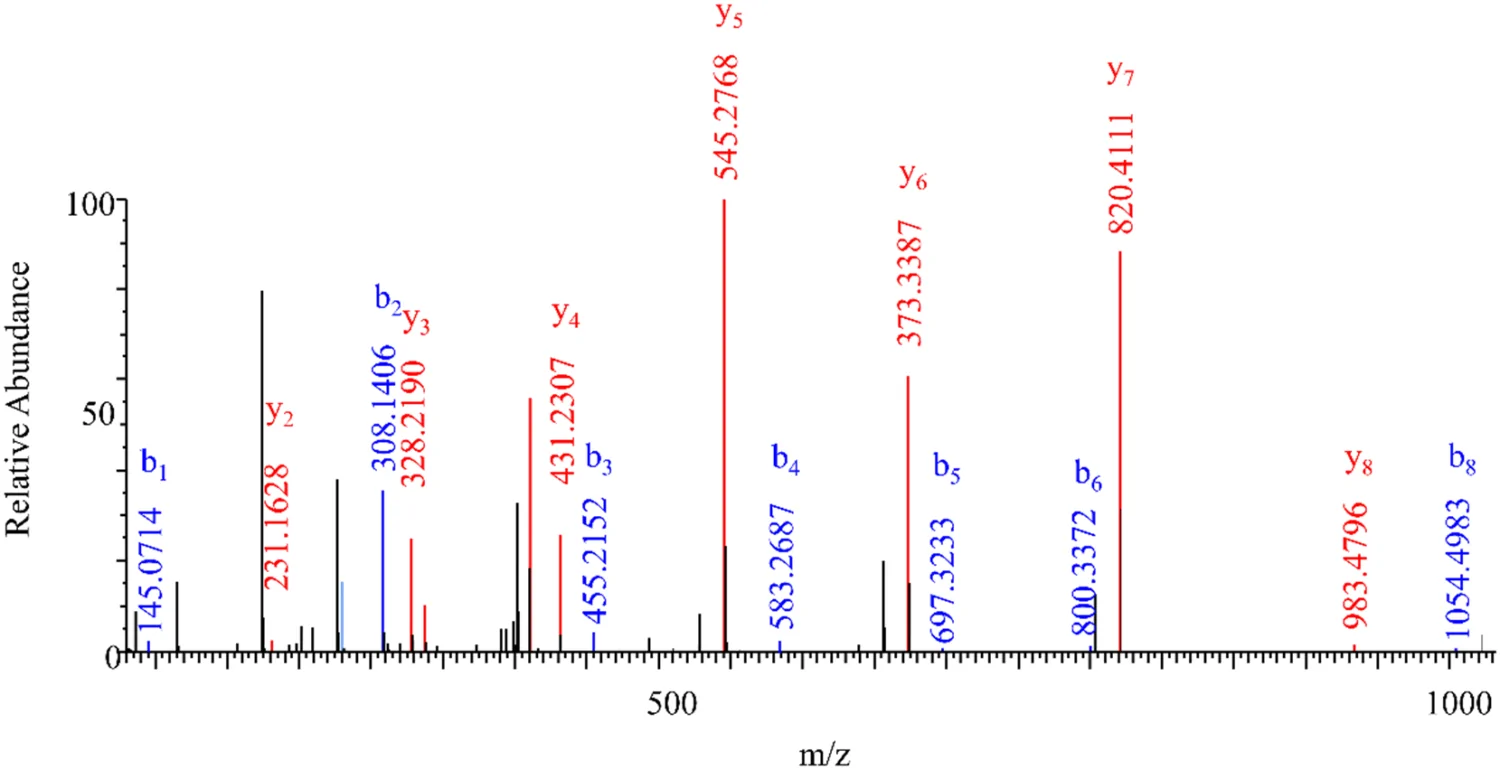
MS/MS spectrum of the target impurity and its corresponding theoretical b and y fragment ions.

Notably, no additional +56 Da species were detected apart from Mpa-*t-*Bu, nor were any secondary fragments observed that would originate from *t-*Bu attachment at alternative nucleophilic sites such as Tyr(2) (y_8_^+^ 1039.514), Cys(6) (y_4_^+^ 729.382), or Arg(8) (y_2_^+^ 287.219).^4^ Structural assignments corresponding to the diagnostic fragment ions (Tyr(2), Cys(6), and Arg(8)) are provided in the Supporting Information (Figures S5-S7). This outcome is clearly inconsistent with the expected signature of “nonselective carbocation capture” (see Scheme 1).

**Scheme 1:**
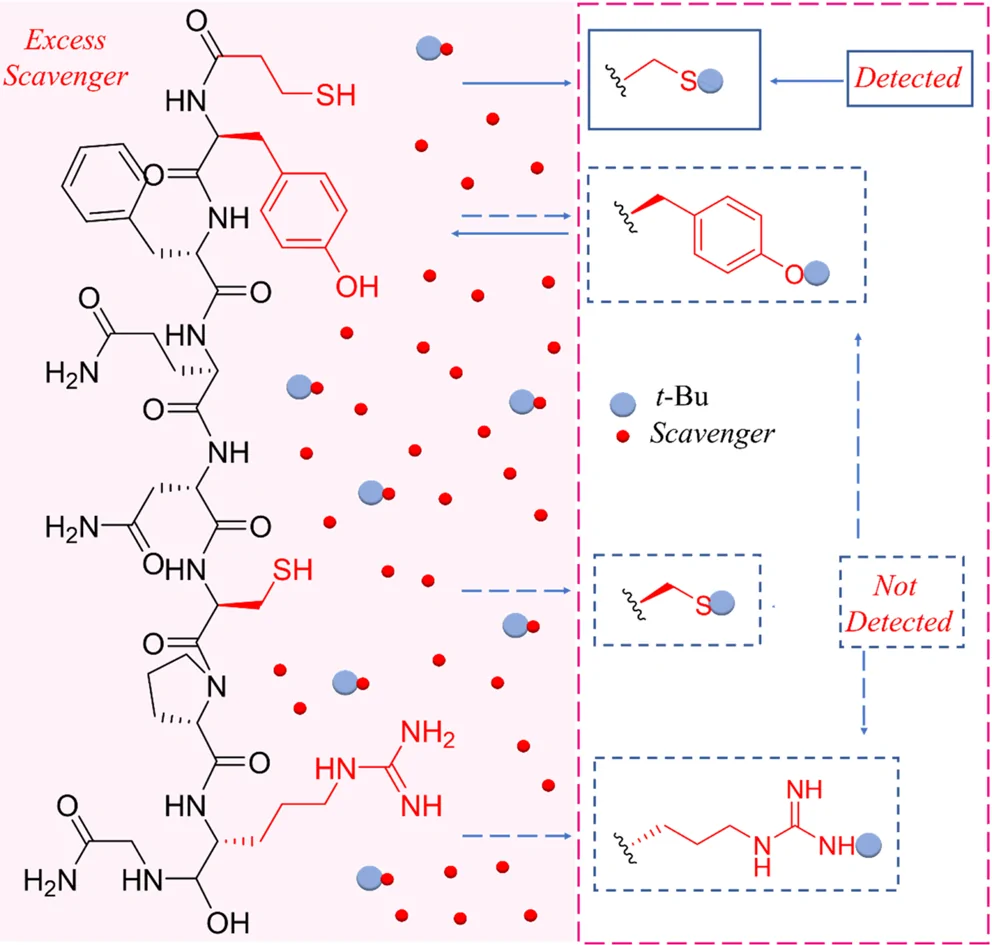
Classical Carbocation-Intermediate-Type Deprotection Pathway Lacks Positional Selectivity and is Expected to Generate Comparable Levels of Mpa­(*t-*Bu), Cys­(*t-*Bu), and Arg­(*t-*Bu) Alkylation Products^a^

a However, the corresponding diagnostic MS/MS fragment ions were not detected, which is inconsistent with a carbocation intermediate-driven mechanism.

Repeated analyses across multiple batches consistently demonstrated a high degree of positional selectivity for this impurity, further confirming that it does not arise from incomplete deprotection of the Tyr(2) *tert*-Bu group. At the same time, such strict site specificity contrasts sharply with the stochastic alkylation typically associated with a classical carbocation intermediate pathway.This pronounced site selectivity cannot be readily explained by a diffusion-controlled mechanism and, therefore, motivates the consideration of an alternative, intramolecular reaction pathway.

### 3.4 Proposed Mechanism of Impurity Formation: Vicinal-Thiol-Induced Intramolecular Migration

Based on the above evidence, we propose that the impurity most plausibly arises from a vicinal-thiol-induced intramolecular migration pathway, which is consistent with the experimental observations and competes with the classical carbocation intermediate pathway. This mechanistic hypothesis is supported by three structural and chemical considerations:

(1) The weak protonation propensity of the tyrosine phenolic oxygen disfavors a carbocation intermediate pathway.
  In *t-*Bu–O–Tyr, the phenolic oxygen is conjugated with the aromatic ring, leading to partial delocalization of its lone pair. As a result, its proton affinity in TFA is significantly weaker than that of aliphatic alcohols, reducing the likelihood of facile C–O bond cleavage and diminishing the formation of a *t-*Bu^+^. The chemoselectivity of modern coupling reagents heavily favors amide-bond formation. With the carbocation intermediate pathway disfavored, the system is more likely to proceed through alternative, mechanistically feasible competing reactions.
(2) Spatial proximity between the Mpa thiol and Tyr­(*t*-Bu) enables an intramolecular nucleophilic attack.
  Within the structure of desmopressin, the thiol group of Mpa(1) lies in close spatial proximity to the O-*t-*Bu moiety of Tyr(2)­(*t-*Bu). The predicted 3D structure showing the spatial proximity of the thiol and t-Bu-O-Tyr is provided in the Supporting Information (Figure S8). This geometry provides a highly accessible nucleophilic site and gives rise to a locally elevated effective molarity.^14^ In this process, departure of the phenolic oxygen with the bonding electron pair occurs concomitantly with nucleophilic attack by the thiol sulfur via lone pair donation to the reaction center. As a result, C–O bond cleavage and C–S bond formation proceed in a concerted manner. While the main product is still predominantly formed through diffusion-controlled intermolecular pathways, the probability of intramolecular migration is significantly enhanced, rendering its contribution non-negligible. This vicinal-thiol-induced intramolecular migration proceeds with minimal entropic penalty. As a result, it can effectively compete with diffusion of the reactive intermediate away from the site of generation.
(3) The thiol group, as a “soft” nucleophile, more readily promotes an intramolecular migration in protic, highly polar media
  compared with hydroxyl groups, thiols (−SH) exhibit greater nucleophilic softness and higher polarizability.^15^ In strongly acidic cleavage media, thiols predominantly exist in a neutral yet sufficiently nucleophilic form, enabling them to stabilize the developing transition state during backside attack on the *t-*Bu moiety. In contrast to classical charged nucleophiles such as OH^–^, the neutral thiol can directly approach the Tyr–O–*t-*Bu bond. This process represents a shift from a neutral to a charge-separated state. Protic, highly polar solvents effectively stabilize such charge development, thereby facilitating the intramolecular migration.

This mechanism provides a coherent explanation for the above experimental observations: (1) strict site selectivity; The reaction does not originate from simple competition between reactive side chains and external scavengers for carbocations. Instead, it is governed by a kinetically controlled competition between a conformation-induced intramolecular migration process and escape of the carbocation from the local microenvironment, thereby resulting in pronounced site selectivity (2). The slight increase in impurity levels observed under reinforced deprotection conditions (e.g., an elevated temperature and prolonged reaction time) can be rationalized by a greater fraction of molecules overcoming the activation barrier associated with the intramolecular transfer process. This behavior cannot be adequately explained by a typical diffusion-controlled intermolecular quenching mechanism.

### 3.5 Validation of the Proposed Mechanism:Strengthening the Scavenger Conditions Failed to Eliminate the Impurity

According to the proposed mechanism, under conditions where the scavenger is present in a large excess, carbocation intermediates that successfully escape the intramolecular microenvironment predominantly undergo intermolecular quenching to form the main product. In contrast, impurity formation arises primarily from those carbocations that fail to escape and instead undergo an intramolecular migration. Therefore, the impurity level is fundamentally governed by the probability of intramolecular capture of the carbocation immediately after its generation, which is largely dictated by the molecular spatial arrangement and local conformational preorganization. Under such conditions, further strengthening of the scavenger conditions (e.g., increasing scavenger loading or reaction severity) should not be expected to significantly alter this partitioning and thus has minimal impact on the impurity level.

Carbocation-intermediate-type pathways are highly sensitive to the composition and strength of the scavenger system. If a substantial amount of *t-*Bu^+^ were indeed participating in electrophilic attack, then increasing the equivalents of scavengers or introducing more nucleophilic thiol-based traps (such as EDT or 3-Mpa) should markedly suppress the formation of the byproduct.^16^

To validate our proposed mechanism, we tested several strategies, including varying the ratios of scavengers and expanding the set of nucleophilic trapping reagents. The resulting crude-product profiles after these optimized treatments are shown in Figure 4 and summarized in Table 2. The corresponding raw chromatograms are provided in the Supporting Information (Figures S9–S12).

**Figure 4:**
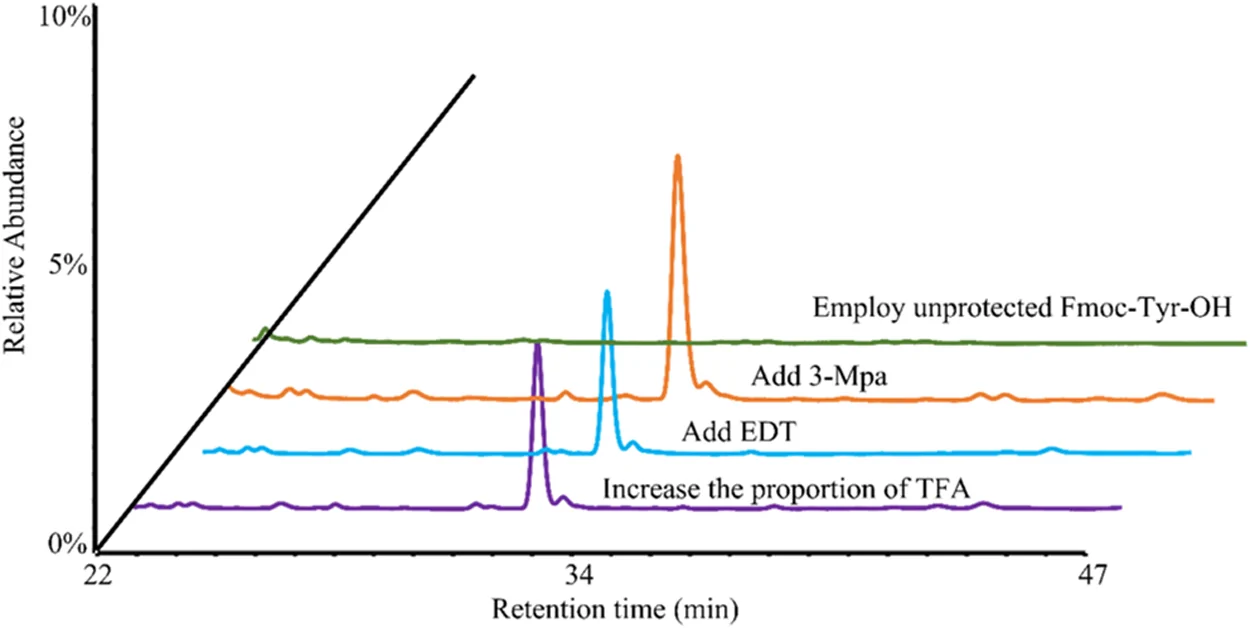
Comparative chromatographic profiles of the crude peptide obtained after process modifications, including added EDT, added 3-mercaptopropionic acid (3-Mpa), varied scavengers’ ratio and employed unprotected Fmoc-Tyr-OH.

**Table 2:** Impurity Content and Crude Peptide Purity in Strengthening Scavenger Conditions

proportion of components	change	Mpa(*t*-Bu) impurity content (%)	crude peptide purity (%)
TFA/TIS /TAO/PhOH/H_2_O = 80/8/4/4/4+DTT(2eq)+Tcep(1eq)	vary scavengers’ ratios	4.73%	85.7
TFA/TIS/TAO/3-Mpa/PhOH/H_2_O = 80/6/4/4/4/2+DTT(2eq)+Tcep(1eq)	Add 3-Mpa	5.08%	82.5
TFA/TIS/TAO/EDT/PhOH/H_2_O = 80/8/3/3/3/3+DTT(2eq)+Tcep(1eq)	Add EDT	4.14%	86.5

In the validation studies, particular attention was given to EDT and 3-MPa. EDT is widely recognized as an efficient scavenger for carbocationic species, especially in protecting thiol functionalities, whereas 3-Mpa can likewise compete for highly reactive cationic intermediates through its thiol group, thereby safeguarding the thiol on Mpa(1) from electrophilic attack. Consequently, if the classical carbocation intermediate pathway was operative, incorporation of these trapping reagents should substantially reduce the likelihood of *t-*Bu^+^ reacting with thiols to generate alkylated byproducts. However, the impurity remained well above the typical reporting threshold for peptide-related impurities (0.5%) and continued to significantly impair overall yield. These findings further demonstrate that the impurity is not formed through a carbocation intermediate-type nonselective *t-*Bu alkylation, and the available validation experiments are inconsistent with a carbocation intermediate pathway and validate our mechanism.

### 3.6 Mechanism-Driven Process Optimization: From “Stepwise Deprotection” to “Upstream Avoidance”

On the basis of the proposed reaction mechanism, a hierarchical evaluation of potential process-engineering strategies was undertaken to suppress impurity formation, including stepwise deprotection, ensuring that tyrosine deprotection occurs before liberation of the Mpa thiol, thereby preventing the coexistence of a free thiol with *t-*Bu-protected Tyr. Two potential approaches can be envisioned: (1) replacing the Tyr protecting group with a more labile one (e.g., BrZ) and (2) substituting the Mpa protecting group with a more robust variant (e.g., Acm).^17^

However, introducing an additional deprotection step would expose the peptide to new acidic or basic conditions, extend the overall synthesis, increase solvent and reagent consumption, and increase the risk of incomplete deprotection or undesired side reactions. In solving one problem, such modifications may inadvertently introduce new challenges, including an elevated side-reaction risk, greater process complexity, and increased manufacturing cost.

Thus, although these strategies are theoretically viable, they should be regarded as options of last resort to be employed only when alternative process solutions prove insufficient.

It was essential to reassess the actual necessity of employing a *t-*Bu protecting group on the Tyr side chain. In the original synthetic design, this protection was introduced to prevent undesired esterification between the phenolic hydroxyl and activated carboxylate intermediates during subsequent coupling steps.^2^ However, under standard SPPS conditions, amide-bond formation overwhelmingly outcompetes ester formation: the nucleophilicity of the α-amino group is substantially higher than that of the phenolic hydroxyl, and commonly used coupling reagents generate activated esters that intrinsically favor reaction with amines.^18^ Although the use of unprotected Fmoc-Tyr-OH has been described, prior reports^19–23^ are confined to specialized sequences or nonstandard reaction conditions (e.g., specific solvents or coupling reagents). Its application in a commercial, platform-compatible synthetic process for desmopressin remains unreported.

From an electronic standpoint, the lone pair on the Tyr phenolic oxygen is delocalized into the aromatic ring, further reducing its nucleophilicity and diminishing its propensity to undergo side reactions with activated carboxyl groups. Moreover, Tyr occupies the second position from the N-terminus in the target peptide, which means that only one downstream coupling step remains. This greatly limits both the exposure time and the cumulative probability of any phenolic side reactions. Collectively, these factors suggest that the omission of the *t-*Bu group on Tyr is theoretically safe. In addition, it may be preferable for this specific sequence.

Guided by this rationale, we evaluated the use of unprotected Fmoc-Tyr-OH as a starting amino acid. Experimental outcomes confirmed that this modification did not generate any phenol-related byproduct. More importantly, it eliminated the structural prerequisite for the intramolecular *tert*-Bu migration, thereby completely suppressing the +56 Da impurity. Analytical data demonstrated that the *t*-Bu migration species was entirely absent, and the crude purity increased significantly to 93.5%. These findings highlight that removing an unnecessary side-chain protecting group not only prevents the atypical migration pathway but also simplifies the synthesis and improves both the product quality and process robustness.

## 4 Conclusions

This study provides a comprehensive structural and mechanistic analysis of a persistent site-selective *tert*-Bu migration impurity in the synthesis of desmopressin. By integrating high-resolution MS/MS characterization with systematic process stress tests, we demonstrated that this impurity is not consistent with canonical incomplete deprotection or a diffusion-controlled carbocation intermediate alkylation pathway. Instead, it most plausibly arises from a vicinal-thiol-induced intramolecular migration, a pathway facilitated by the specific spatial proximity of the Mpa and Tyr residues. Further strengthening of the scavenger condition experiments validates our proposed mechanism.

This study does not merely identify a process impurity, but reveals a proximity-governed selectivity, highlighting that intramolecular spatial arrangement can significantly bias carbocation capture pathways. These findings highlight a critical oversight in standard protecting group strategies: sequence-specific geometry can override general reactivity trends. While *tert-*butyl cleavage in peptide synthesis is generally described as a nonselective carbocation intermediates process, the present study reveals that such reactions can exhibit pronounced site selectivity when governed by intramolecular proximity. This observation challenges the conventional assumption of stochastic carbocation capture and highlights the importance of sequence-dependent spatial organization in directing side reactions. The findings provide a new perspective on impurity formation in peptide synthesis and emphasize the need to consider local geometry, in addition to intrinsic reactivity, in protecting group design. For peptides containing thiol residues adjacent to *t-*Bu-protected side chains, the risk of intramolecular migration is significant. We validated a mechanism-based solution—the use of unprotected Fmoc-Tyr-OH—which proved to be a robust, scalable, and cos*t-*effective method to eliminate this impurity. This work establishes a clear precedent for ″structure-mechanism-process-verification″ optimization, offering a valuable reference for controlling atypical side reactions in complex peptide synthesis.

## Supplementary Material



## Notes

Synthesis of desmopressin acetate, raw chromatograms of the crude peptide, structural assignments corresponding to the diagnostic fragment ions, and predicted 3D structure (PDF)
