# Supplementary material for: Tyr–Mpa Proximity-Induced Atypical tert-Butyl Intramolecular Migration in Desmopressin: Mechanistic Elucidation of a Site-Selective Impurity and Its Suppression Strategy
Source: ACS Omega. 2026 Aug 27;11(35):53469–76. doi: 10.1021/acsomega.6c07280 (PMC13647234; doi:10.1021/acsomega.6c07280)
Supplement: Supplementary file 1 [file ao6c07280_si_001.pdf]

Original Article

Title Page

Title： Tyr–Mpa Proximity-Induced Atypical tert-Butyl Intramolecular Migration in Desmopressin: Mechanistic Elucidation of a Site-Selective Impurity and Its Suppression Strategy

Author： Xin Lu [a], Fangfang Yuan [a], Xiaojie Han [a], Tingting Qin [a], Qianqian Wang [b], Zengli Li [c], Chuanjun Ge [c], Wenqing Shi [c], Haijiao Bai [a*], Pei Wang [a*]

Affiliations: [a] *Tianjin Institute for Drug Control, Tianjin 300070, China;* [b] *State Key Laboratory of Medicinal Chemical Biology, Nankai University, Tianjin 300350, China;* [c] *Anhui Anke Biotechnology (Group) Co., Ltd, Hefei 230088, China*

Corresponding author: ritaw_81@163.com

Haijiao Bai and Pei Wang are co-corresponding authors and contributed equally to this article.

Materials and methods

Synthesis of Desmopressin Acetate

Preparation of Desmopressin Peptidyl Resin

Desmopressin was assembled on Rink amide–AM resin using standard Fmoc-based solid-phase peptide synthesis (Fmoc-SPPS). Rink amide–AM resin (2.183 g) was charged into a reaction vessel and swollen in DMF (20 mL) for 20 min. After swelling, the solvent was drained and the resin was washed twice with DMF (20 mL each). Fmoc deprotection was carried out by treatment with 20% piperidine in DMF (20 mL) for 20 min. The deprotection solution was drained, and the resin was washed six times with DMF. For coupling of the first amino acid, Fmoc-Gly-OH (1.213 g) and HOBt (0.664 g) were dissolved in DMF (15 mL) and the solution was cooled to 0–15 ℃. DIC (0.8 mL) was then added, and the mixture was preactivated for 5–10 min. The activated solution was transferred to the reaction vessel, followed by an additional 5 mL of DMF. Coupling was allowed to proceed for 90 min at 30 ℃ (this temperature was maintained for all subsequent coupling reactions). After completion, the reaction mixture was drained and the resin was washed three times with DMF. Completion of coupling was confirmed by a Kaiser test, which was negative (colorless), indicating full acylation. To cap any unreacted amino groups, the resin was treated with DMF (20 mL), acetic anhydride (1.0 mL), and N-methylmorpholine (NMM, 1.2 mL), and the mixture was gently agitated under a nitrogen stream for 20 min. The solution was drained, and the resin was washed three times with DMF. Fmoc deprotection was then performed using 20% piperidine in DMF (20 mL) for 20 min. The solution was removed, and the resin was washed six times with DMF.

For coupling of the second residue, Fmoc-D-Arg(Pbf)-OH (2.552 g) and HOBt (0.652 g) were each dissolved in DMF (15 mL), cooled to 0–15 ℃, and activated with DIC (0.8 mL) for 5–10 min. The activated solution was added to the reaction vessel, followed by an additional 5 mL of DMF. Coupling was carried out for 90 min at 30 ℃. After completion, the resin was drained and washed three times with DMF. Coupling efficiency was again verified by a Kaiser test, which was negative. Fmoc deprotection was performed using 20% piperidine in DMF (20 mL) for 20 min, followed by drainage and six washes with DMF. Subsequently, the remaining amino acids of the desmopressin sequence were sequentially coupled to the resin using the same activation and coupling protocol described above, affording the fully protected peptidyl resin. The Fmoc-protected amino acids were introduced in the following order:Fmoc-Pro-OH, Fmoc-Cys(Trt)-OH, Fmoc-Asn(Trt)-OH, Fmoc-Gln(Trt)-OH, Fmoc-Phe-OH, Fmoc-Tyr-OH, and Mpa(Trt)-OH.

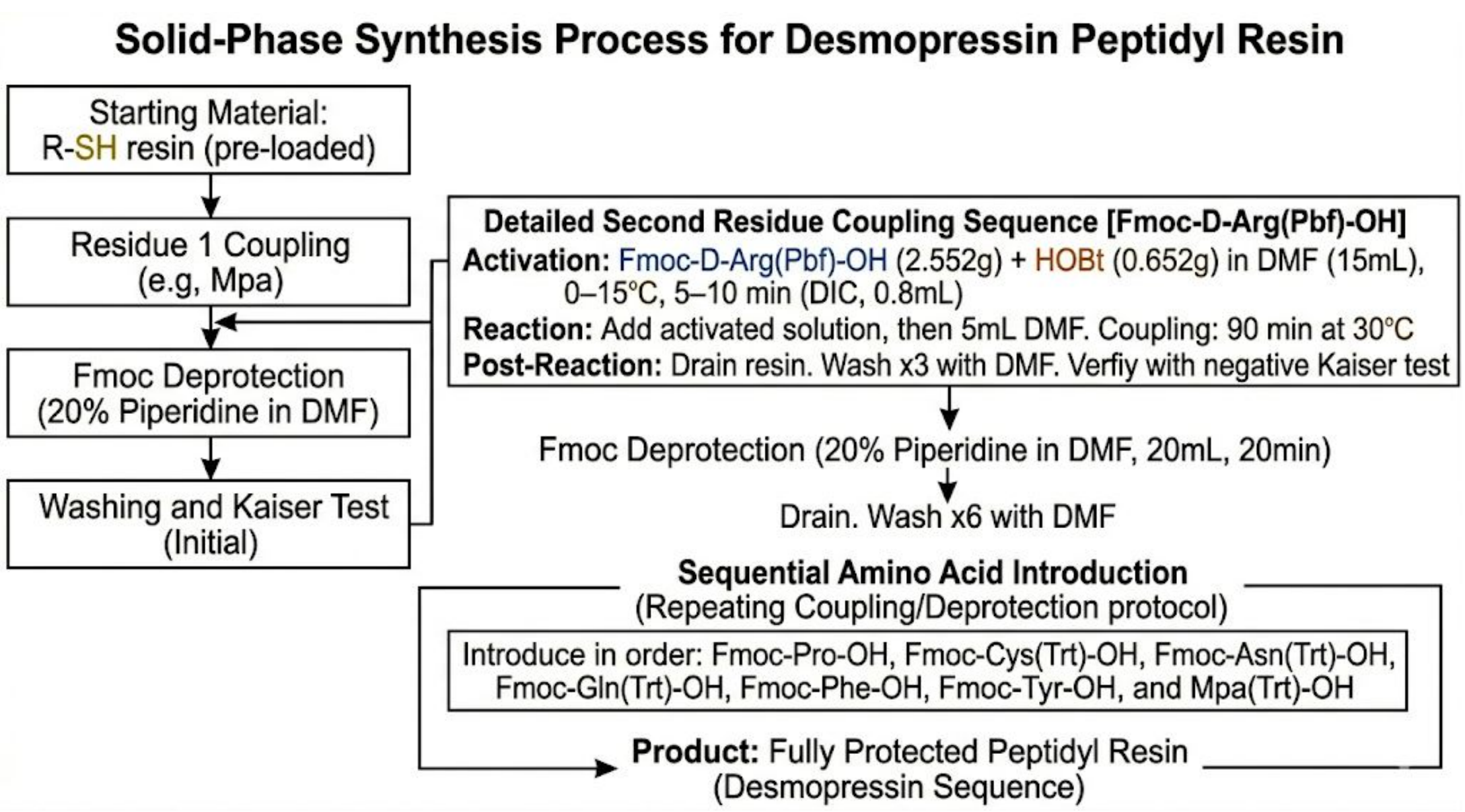

Fig. S1 Complete process flow diagram

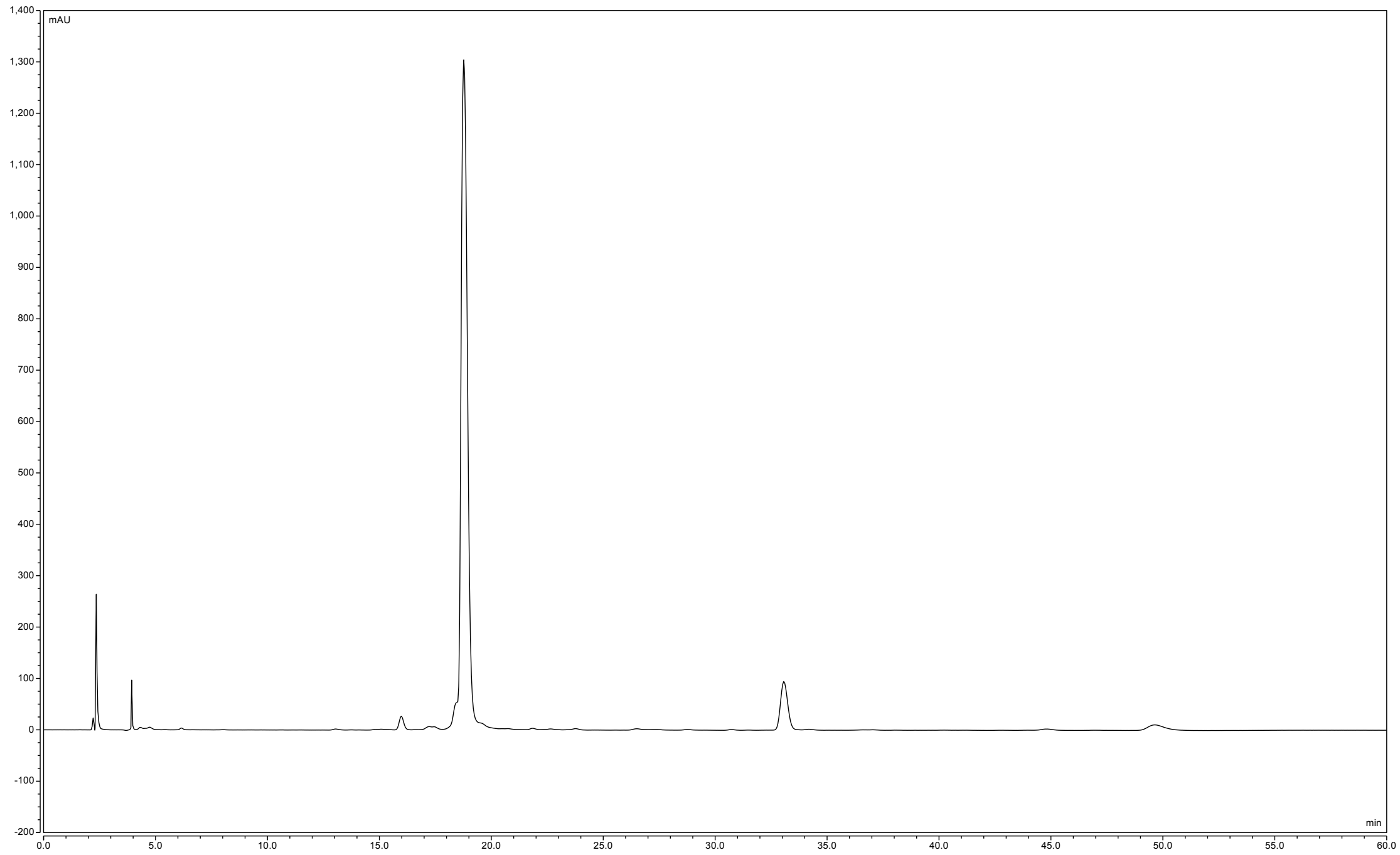

Fig. S2 Raw chromatograms of the crude peptide obtained after increasing TFA content from 80% to 90%.

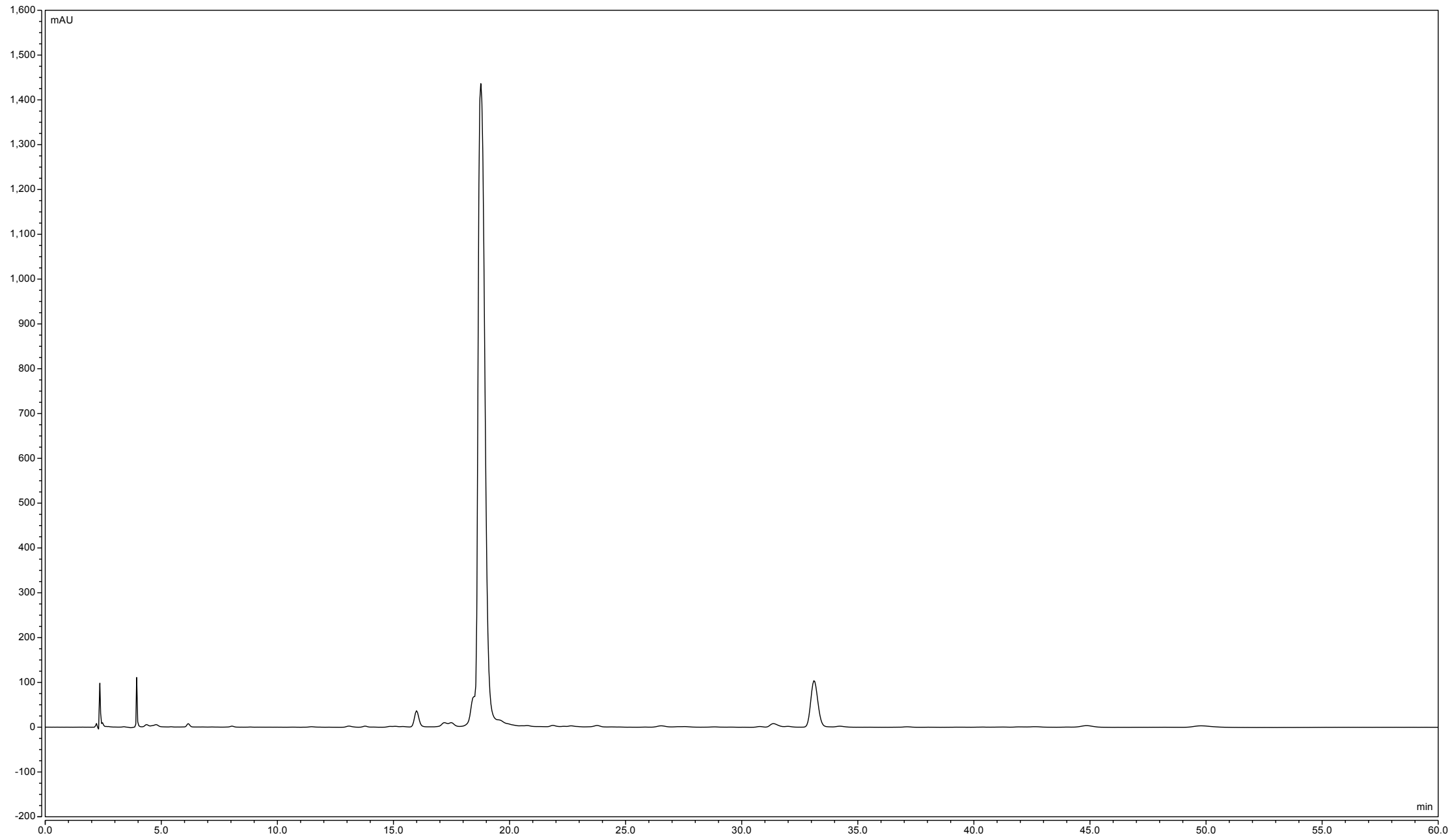

Fig. S3 Raw chromatograms of the crude peptide obtained after elevating reaction temperature from 25℃ to 30℃.

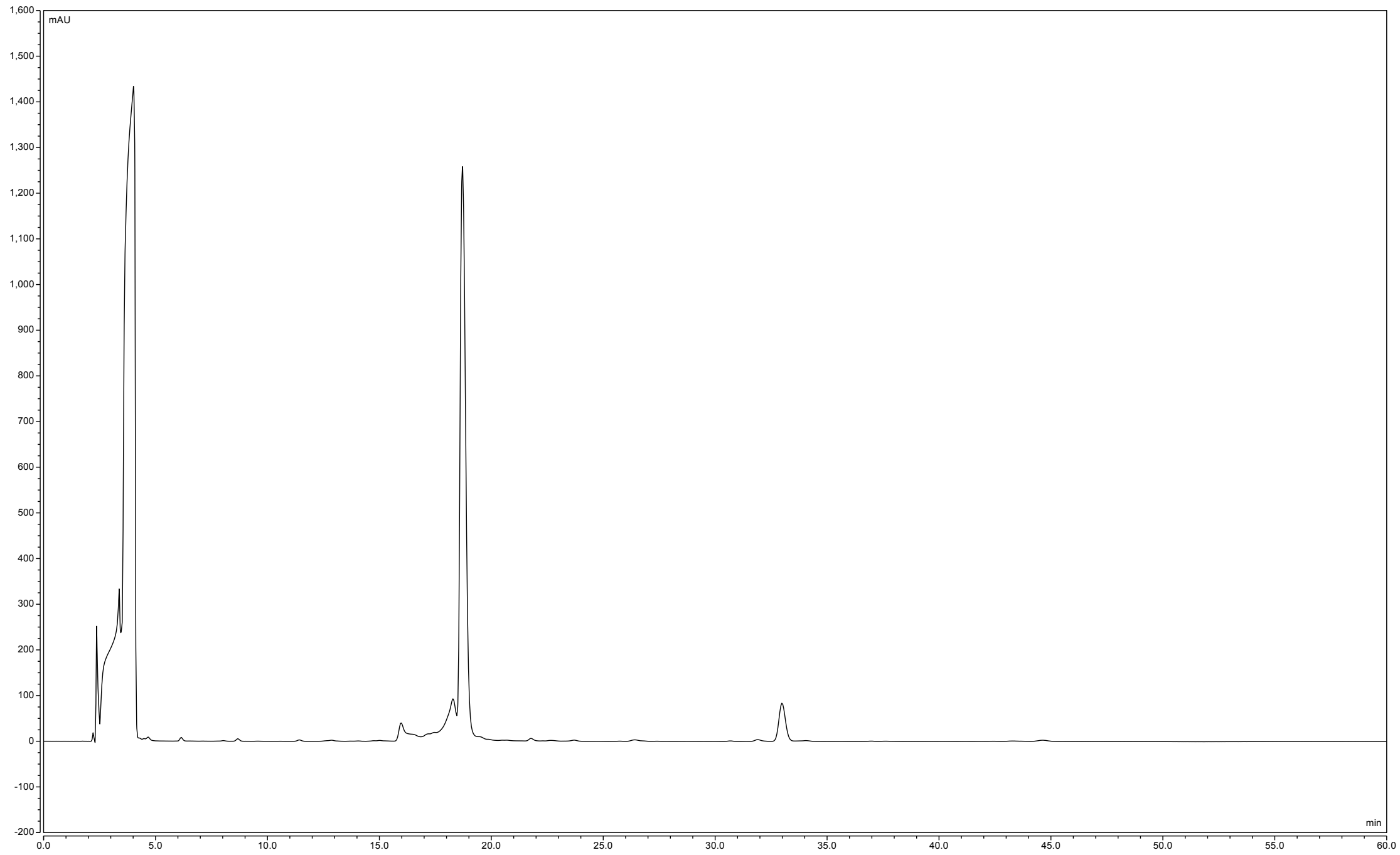

Fig. S4 Raw chromatograms of the crude peptide obtained after extending reaction time from 2h to 2.5h.

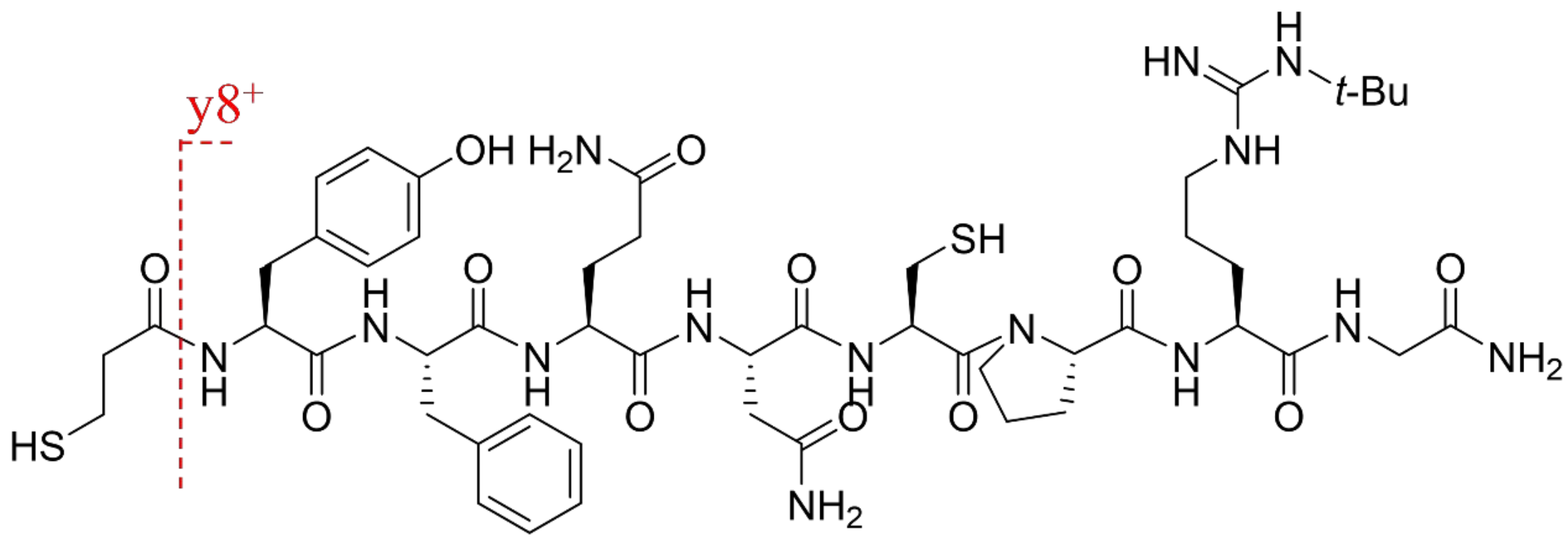

Fig. S5 Structural assignments corresponding to the diagnostic fragment ions originated from t-Bu attachment at Tyr(2)

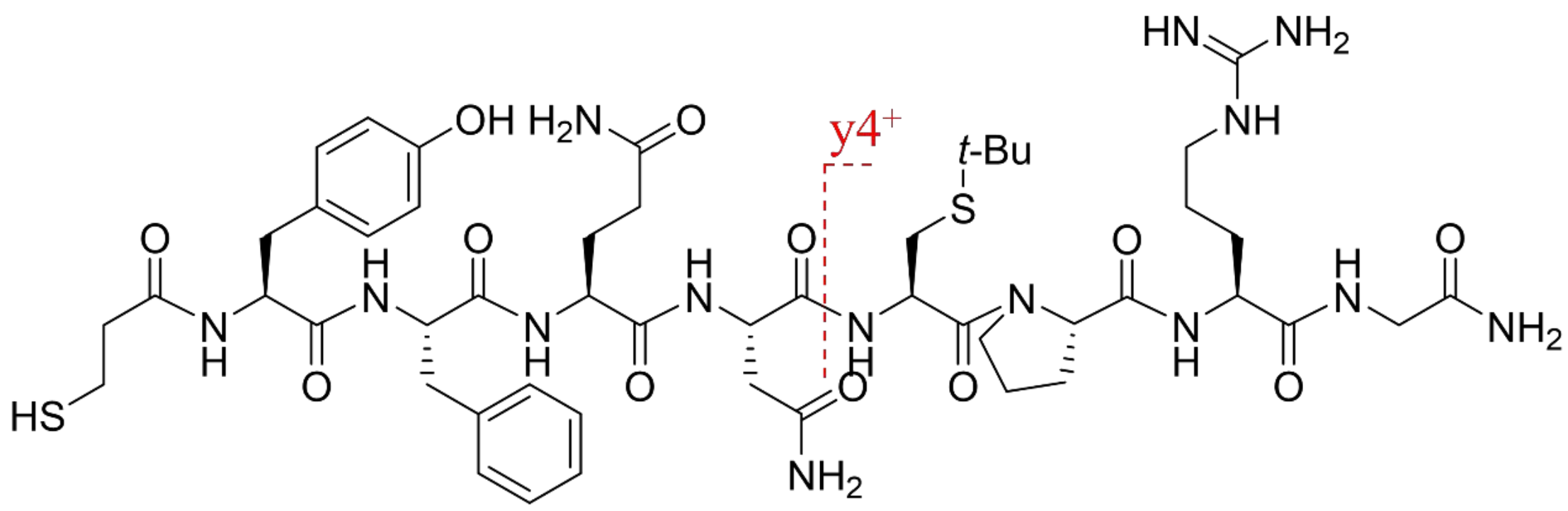

Fig. S6 Structural assignments corresponding to the diagnostic fragment ions originated from t-Bu attachment at Cys(6)

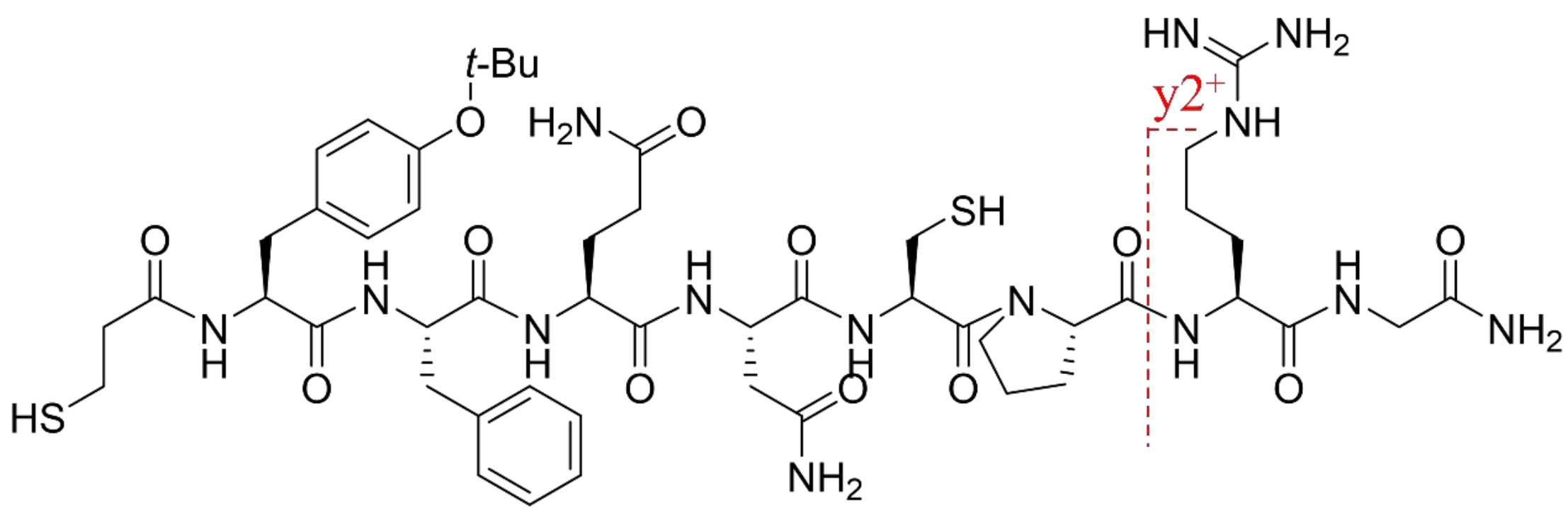

Fig. S7 Structural assignments corresponding to the diagnostic fragment ions originated from t-Bu attachment at Arg(8)

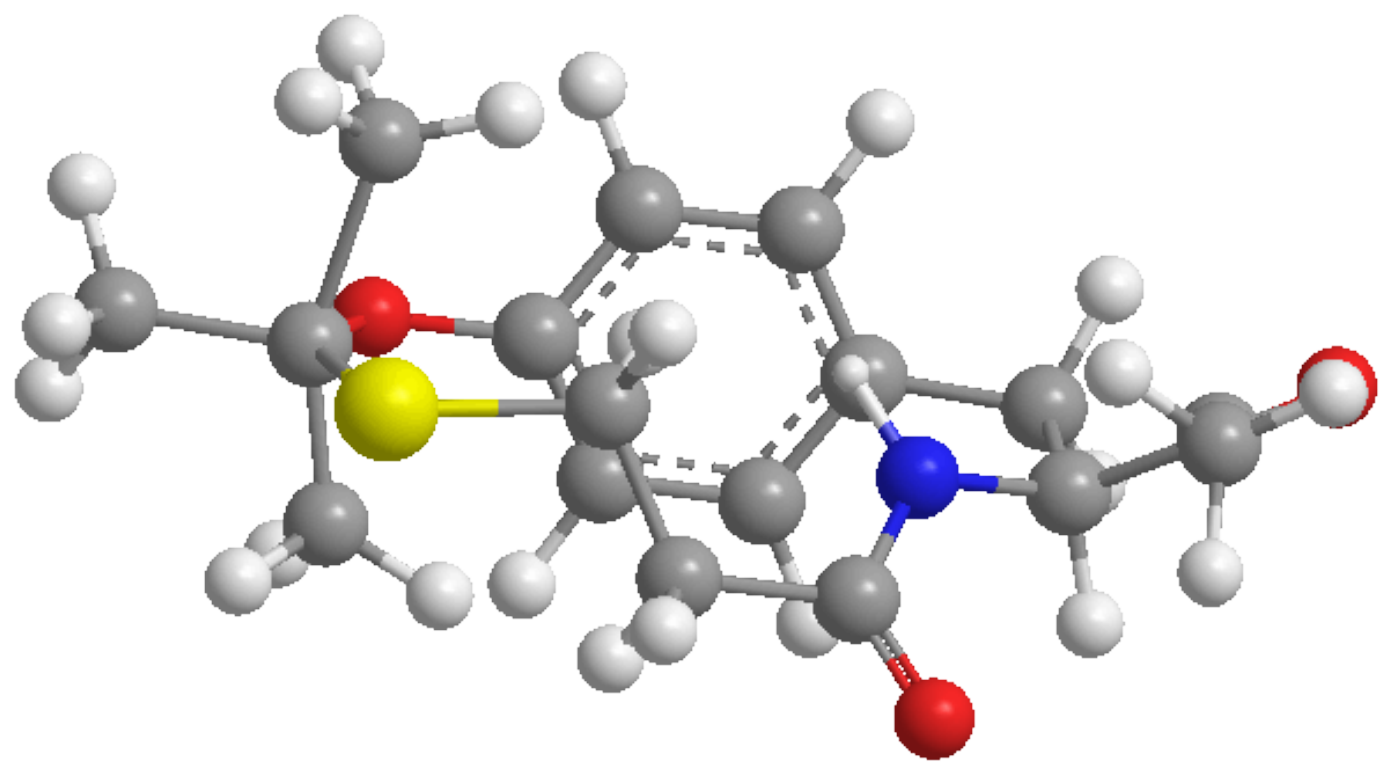

Fig. S8 The predicted 3D structure showing the spartial proximity of the thiol and t-Bu-O-Tyr

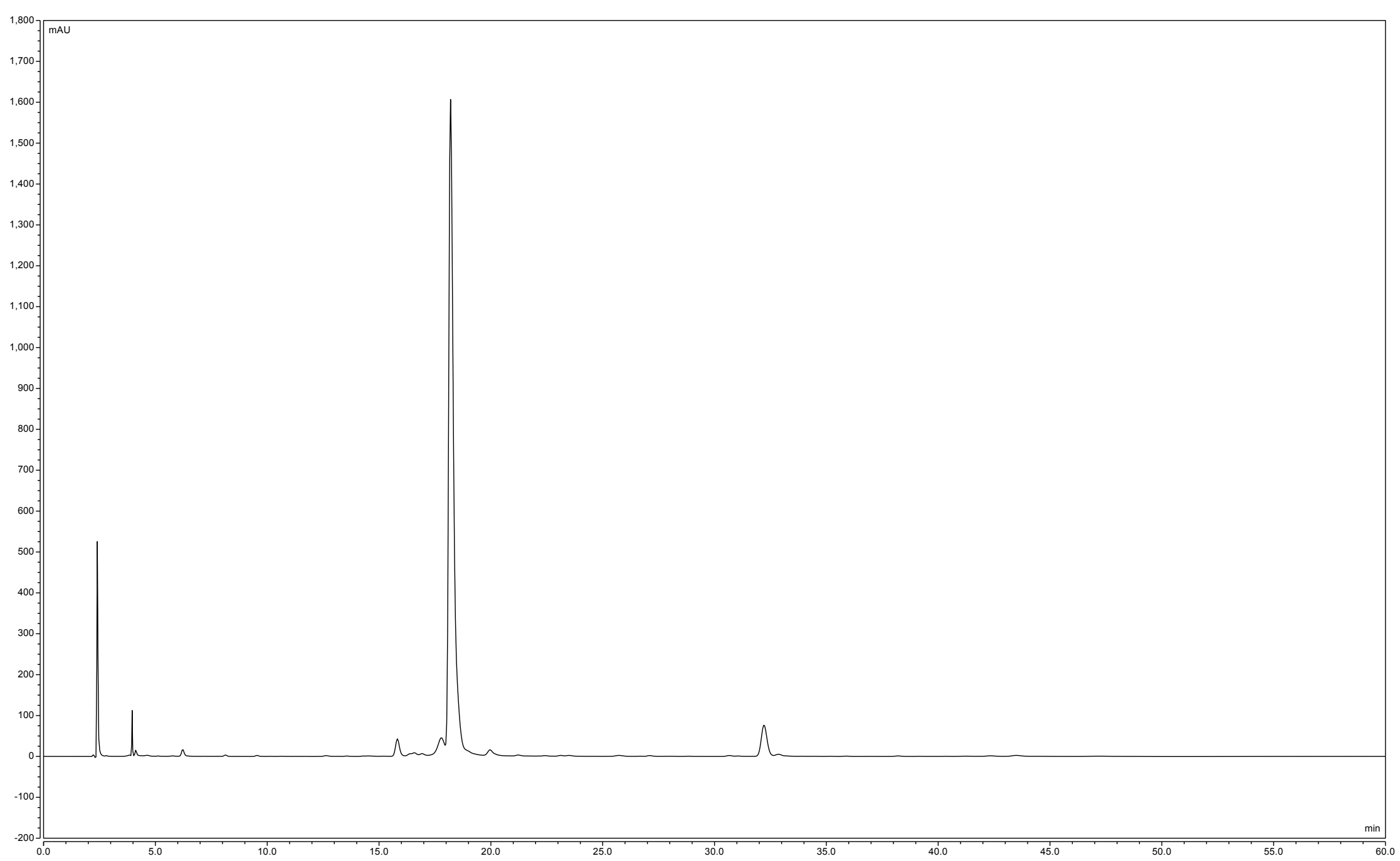

Fig. S9 Raw chromatograms of the crude peptide obtained after adding EDT.

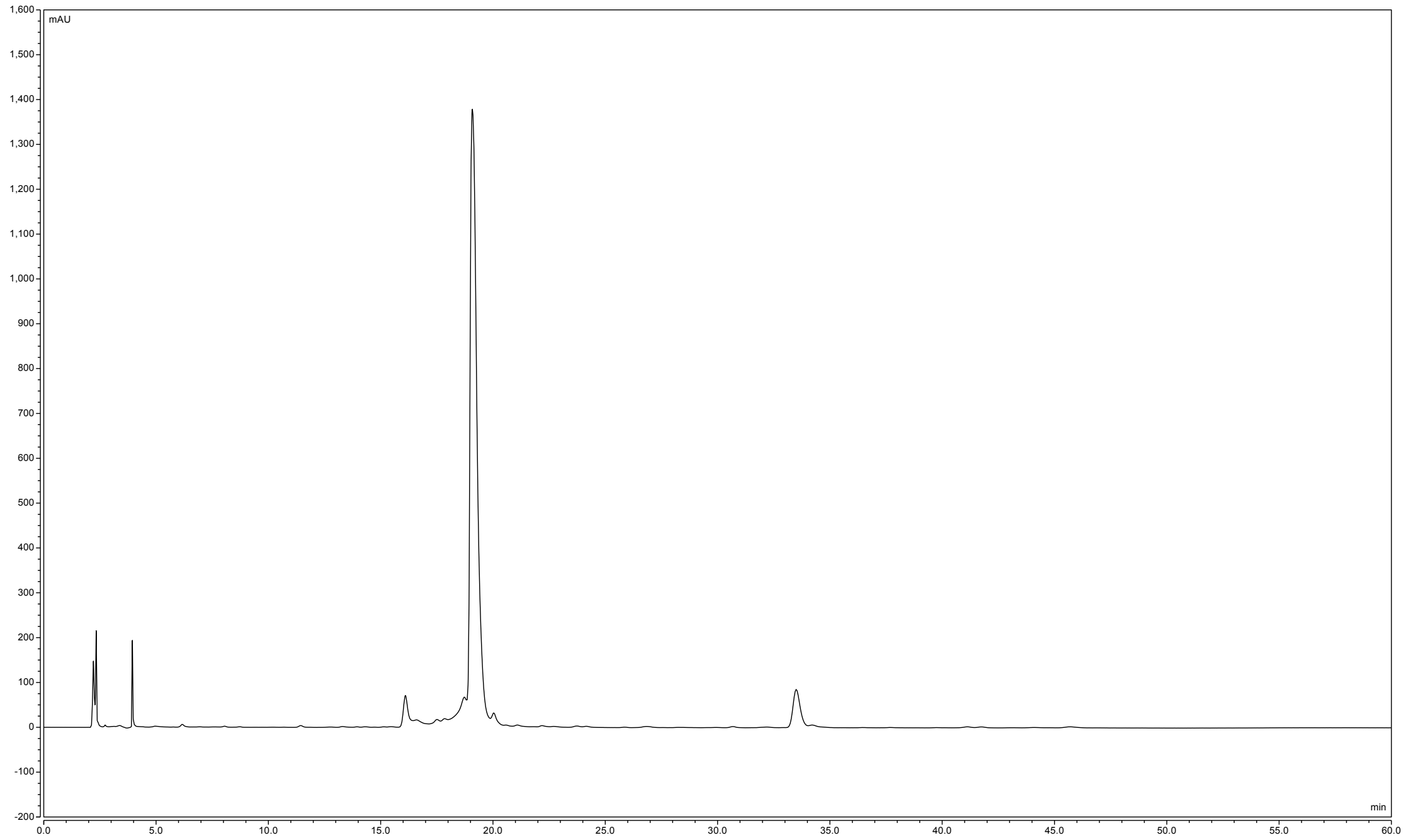

Fig. S10 Raw chromatograms of the crude peptide obtained after adding 3-Mpa.

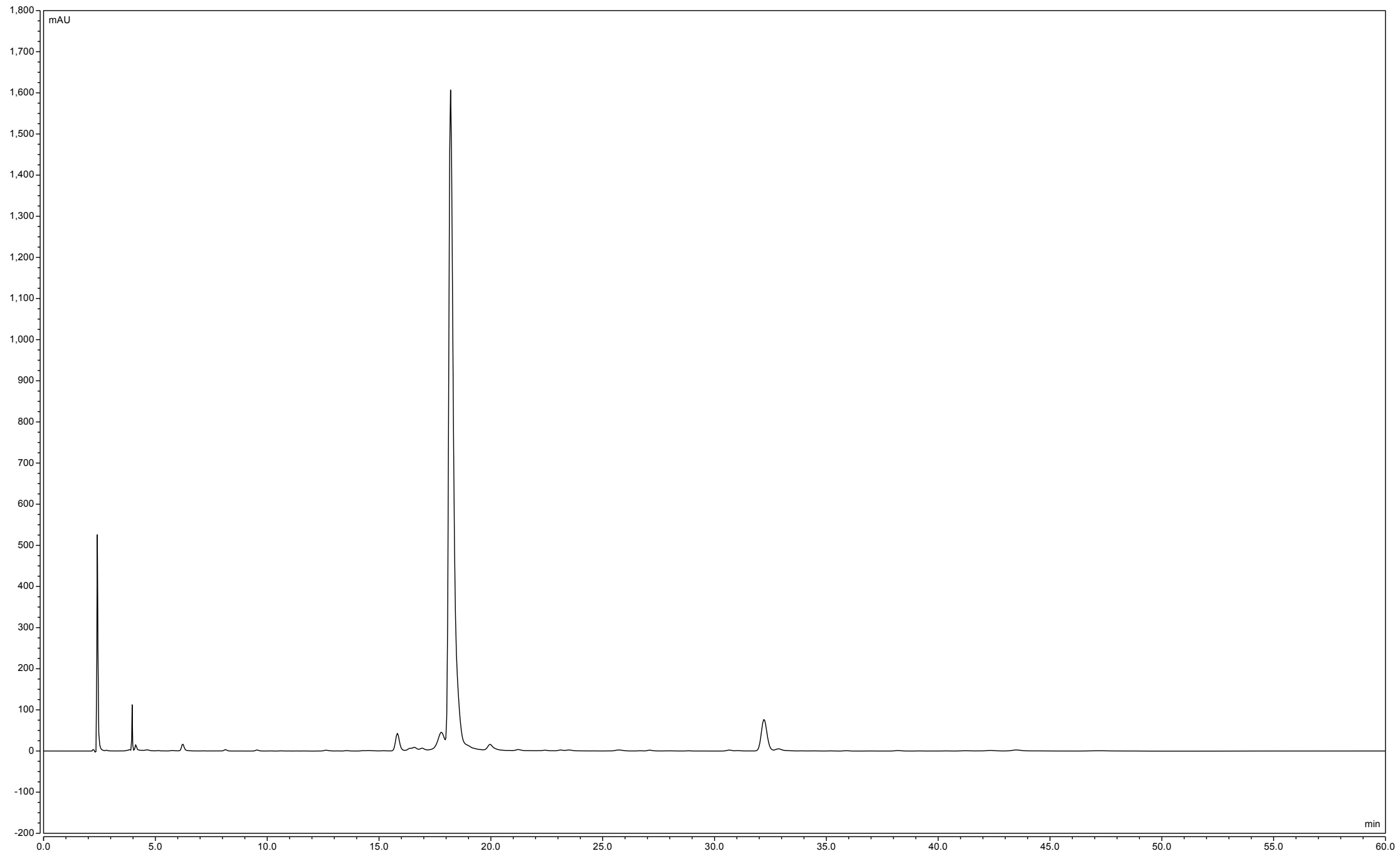

Fig. S11 Raw chromatograms of the crude peptide obtained after varing scavengers’ ratio.

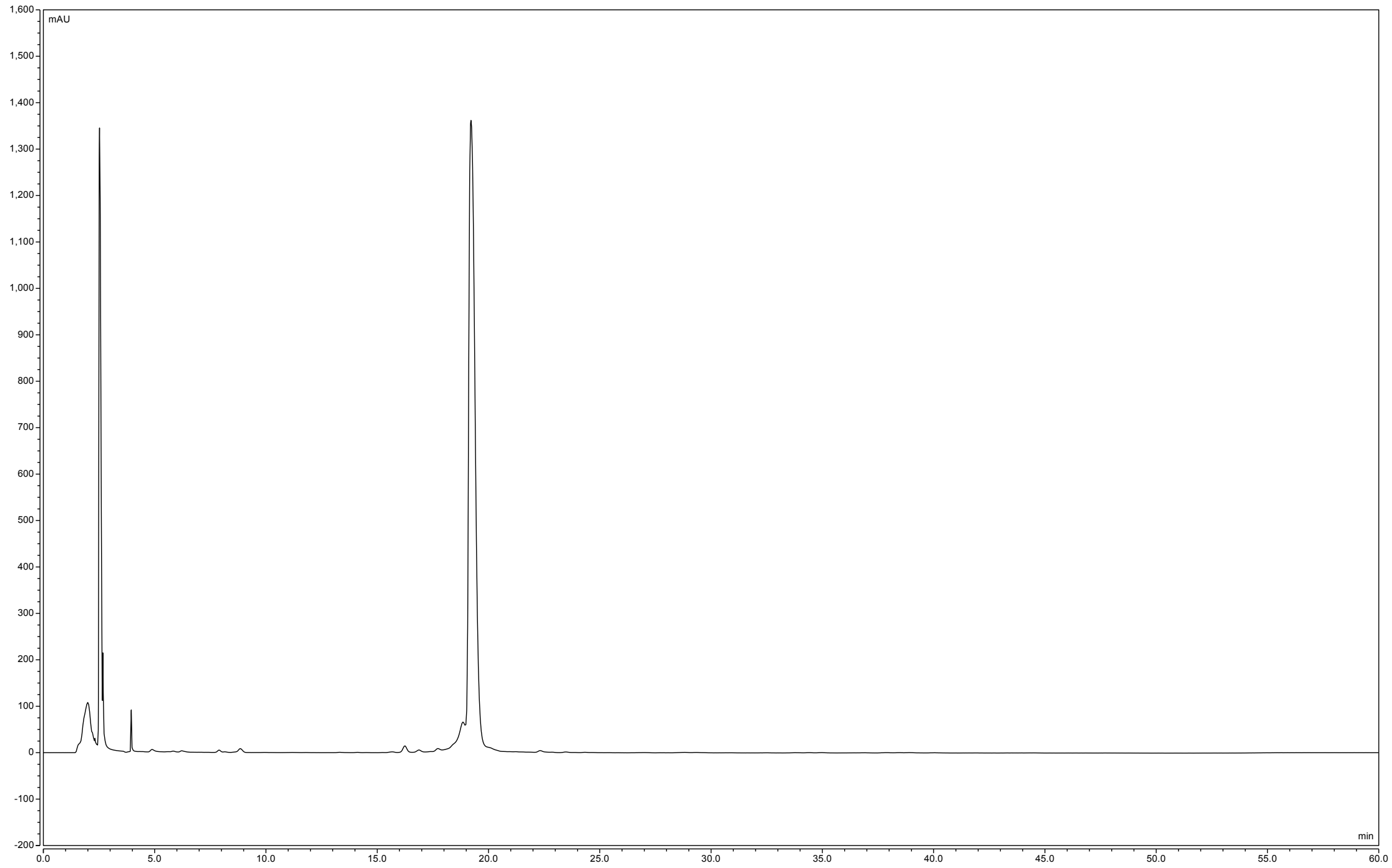

Fig. S12 Raw chromatograms of the crude peptide obtained after employing unprotected Fmoc-Tyr-OH.
